# ‘Sly grog’ and ‘homebrew’: a qualitative examination of illicit alcohol and some of its impacts on Indigenous communities with alcohol restrictions in regional and remote Queensland (Australia)

**DOI:** 10.1186/s13104-017-2691-9

**Published:** 2017-08-01

**Authors:** Michelle S. Fitts, Jan Robertson, Simon Towle, Chris M. Doran, Robyn McDermott, Adrian Miller, Stephen Margolis, Valmae Ypinazar, Alan R. Clough

**Affiliations:** 1 College of Public Health, Medical & Veterinary Sciences, Australian Institute of Tropical Health and Medicine, James Cook University, 1 James Cook Drive, Townsville, QLD 4811 Australia; 2 College of Public Health, Medical & Veterinary Sciences, School of Nursing, Nutrition & Midwifery, James Cook University, PO Box 6811, Cairns, QLD 4870 Australia; 3 Community-based Health Promotion and Prevention Studies Group, College of Public Health, Medical & Veterinary Sciences, Australian Institute of Tropical Health & Medicine, James Cook University (Cairns Campus), PO Box 6811, Cairns, QLD 4870 Australia; 4 Hunter Medical Research Institute, University of Newcastle, Locked Bag 1000, New Lambton Heights, NSW 2305 Australia; 5 Centre for Chronic Disease Prevention, Faculty of Medicine, Health & Molecular Sciences, James Cook University, PO Box 6811, Cairns, QLD 4870 Australia; 6 Indigenous Health, Indigenous Research Network, Griffith University, Brisbane, QLD 4111 Australia; 7 Royal Flying Doctor Service, Faculty of Medicine, Griffith University, Mt Gravatt, QLD 4122 Australia; 8 Griffith University, Mt Gravatt, QLD 4122 Australia

**Keywords:** Alcohol, Alcohol supply controls, Indigenous Australia

## Abstract

**Background:**

Indigenous communities in Queensland (Australia) have been subject to Alcohol Management Plans since 2002/03, with significant penalties for breaching restrictions. ‘Sly grog’ and ‘homebrew’ provide access to alcohol despite restrictions. This paper describes how this alcohol is made available and the risks and impacts involved. In affected towns and communities across a large area of rural and remote Queensland, interviews and focus groups documented experiences and views of 255 long-standing community members and service providers. Using an inductive framework, transcribed interviews were analysed to identify supply mechanisms, community and service provider responses and impacts experienced.

**Results:**

‘Homebrew’ was reportedly manufactured in just a few localities, in locally-specific forms bringing locally-specific harms. However, ‘sly grog’ sourced from licensed premises located long distances from communities, is a widespread concern across the region. ‘Sly grog’ sellers circumvent retailers’ takeaway liquor license conditions, stockpile alcohol outside restricted areas, send hoax messages to divert enforcement and take extraordinary risks to avoid apprehension. Police face significant challenges to enforce restrictions. On-selling of ‘sly grog’ appears more common in remote communities with total prohibition. Despite different motives for involvement in an illicit trade ‘sly grog’ consumers and sellers receive similar penalties.

**Conclusions:**

There is a need for: (a) a more sophisticated regional approach to managing takeaway alcohol sales from licensed suppliers, (b) targeted penalties for ‘sly grog’ sellers that reflect its significant community impact, (c) strategies to reduce the demand for alcohol and (d) research to assess the effects of these strategies in reducing harms.

## Background

For the Indigenous populations in the developed economies of Canada [1], the United States [2] and Australia [3], legal restrictions on alcohol, specifically designed for remote settlements, have been used. Where rigorous evaluations are available [4–8], such targeted interventions have generally shown favourable effects [9], at least initially. Alcohol is a lead cause of the high rates of premature death and avoidable disease, crime, violence and injuries experienced within Australia’s Aboriginal and Torres Strait Islander (Indigenous) communities [10, 11]. Alcohol management plans (AMPs), involving local controls on the types and quantities of alcohol one is permitted to possess and consume, have been the principal measure used in government policies for reducing alcohol-related harms among Indigenous Australians, particularly in remote locations [12, 13]. Evaluation reports of such measures have commonly reported the availability and consumption of alcohol in defiance of controls. These include reports of activities of unlicensed operators who illegally on-sell alcohol purchased from licensed retailers [14–16]; also noted by key academics and community leaders [3, 17, 18]. This illicit alcohol and its on-selling are referred to colloquially, and in these evaluation reports and commentaries, as ‘sly grog’ and the ‘sly grog trade’ respectively. To a seemingly lesser extent, but also in defiance of restrictions, alcohol fermented from locally-available ingredients (‘homebrew’) has been available and this has also drawn attention from legislators over the past decade [19, 20]. More recently, reports from leading Indigenous health agencies have highlighted additional concerns about the selling of this ‘homebrew’ in some communities [21]. The availability, consumption and marketing of these forms of illegal alcohol have clearly added to the significant challenges faced by communities and policy makers to limit alcohol availability and reduce its harms in these populations.

Despite its importance to communities, service providers, enforcement and other government agencies, and the historical importance of alcohol issues for Indigenous Australians generally, we can find no contemporary, published description or systematic analysis of ‘sly grog’ or ‘homebrew’.

In Queensland’s discrete Indigenous communities, AMPs were first implemented from 2002 to 2003, further tightened in 2008, and were being reviewed when this paper was in preparation [22]. Management of illicit alcohol has long been a priority for the Queensland Government [23] and various strategies have been tried. These include tougher licencing conditions on liquor outlets in and around communities and collaborations between police and other government agencies to identify and apprehend ‘sly groggers’ using media campaigns and a centralised anonymous ‘sly-grogging’ hotline [24, 25]. To reduce ‘homebrew’ alcohol, legislation prohibiting the possession of ‘homebrew’ equipment and products in restricted areas in Queensland was introduced [20].

Recently-published analyses of the general effectiveness of Queensland’s AMPs, concluded that initial achievements in reducing violence and improving community amenity have become undermined over time, in particular by the ongoing availability of illicit alcohol and the urgency to consume it [26–28]. The present paper provides a specific focus on this key public health issue. It reveals the structure and operation of the ‘sly grog’ trade and examines the extent to which ‘homebrew’ is available and traded. Some of the harmful impacts perceived and experienced are identified. A descriptive model of the supply and impacts of illicit alcohol is presented and strategies for reducing these are discussed. Implications for Indigenous governance of alcohol controls in Australia are considered.

## Methods

### Setting

**Fig. 1:**
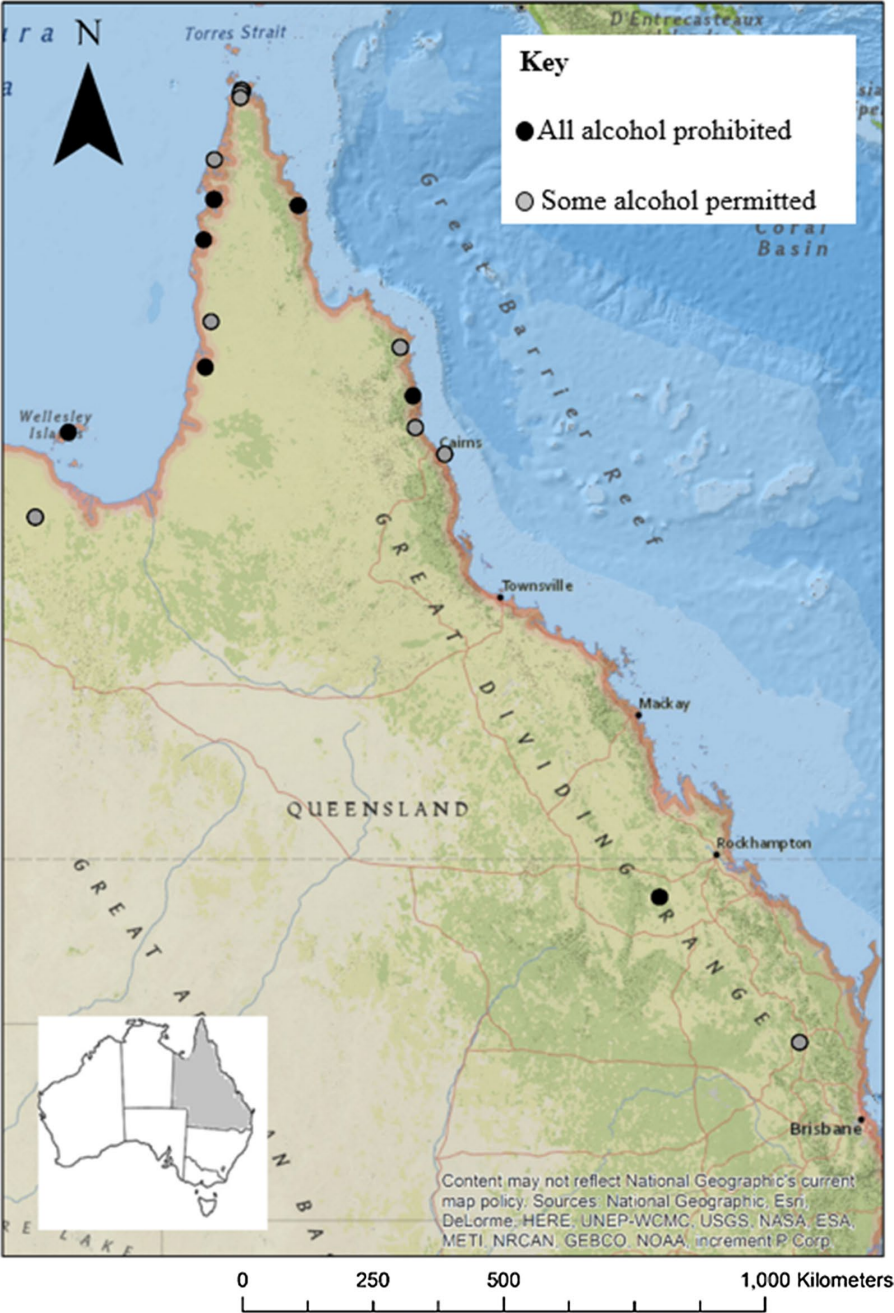
Indigenous communities in Queensland (Australia) with legal controls on alcohol (Alcohol Management Plans). This map was created using ArcGIS^®^ software by Esri. ArcGIS^®^ and ArcMap™ is the intellectual property of Esri and is used herein under license. Copyright © Esri. All rights reserved

The 19 Indigenous communities, situated in 15 Local Government Council areas, where AMPs are in place have been described in detail in previous publications [13, 22]. In summary, they are small, isolated communities comprised of approximately 16,000 Indigenous residents and located mainly in the rural and remote areas of north Queensland (Fig. 1). Two communities are on islands approximately 20 km offshore. Within 5–300 km road distance from the mainland communities, there are several large towns and regional centers where alcohol can be purchased from licensed retailers with few restrictions. When this evaluation study commenced, the residents of eight of the 19 communities were permitted some access to alcohol on a restricted basis, while in the remaining 11 communities all alcohol had been prohibited since 2008. Prior to 2008, there were few practical restrictions on alcohol availability in the 19 communities [8, 13].

### Data collection

Semi-structured interviews were conducted between May 2013 and July 2015 with key stakeholders and service providers in Indigenous communities across north Queensland where these alcohol restrictions are in place, and in the nearby towns and regional centers [27]. Hand-written verbatim notes were made or interviews were audio-recorded where participants gave consent. The semi-structured interview schedule below guided the interviews:

- Do you think the AMPs are working (i.e. have they achieved their aims)?
  - What were the favourable achievements?
  - What were the unfavourable effects?
- What do you think should happen with AMPs in the future?

### Participants

As already described elsewhere [27, 28], key stakeholders and service providers were interviewed about the impacts of AMPs if they were: (1) known by or referred to the research team as highly-regarded and knowledgeable; (2) had lived in or serviced the affected communities and towns in the study region at any time in the years prior to 2009; or (3) had a current or past role in a service with either direct or indirect responsibility for managing the issues and consequences surrounding AMPs in the region. Purposive sampling was used, where participants were selected from agency lists and from those known to be working in these sectors by the research team. Relevant groups included: Elected Local Government Councilors, employees and community Elders, justice and liquor regulation, education and welfare, health, private enterprise, non-government organisations and persons, Indigenous policy and housing and homelessness support groups.

A ‘snowball’ approach was used whereby each participant was asked to recommend other relevant agencies and/or individuals in the region [27]. This ensured a wide spectrum of views on the impact of ‘sly grog’ was captured. Sampling continued until participants recommended no new sectors for interview and until there was some representation from the more remote communities and the other localities nearer regional centers with a balanced representation of Indigenous and non-Indigenous participants.

### Analysis of interview information

From the 382 participants interviewed, a total of 255 participants made more than 542 comments in 196 interviews/focus groups which referenced illegal drinking [27]. The recently-published examination of key stakeholders’ and service providers’ views of the effectiveness of AMP restrictions [27] found that illegal drinking was the most frequently reported issue of concern, one which was seen as potentially undermining the historically significant reductions achieved in violence and injury in these localities.

**Fig. 2:**
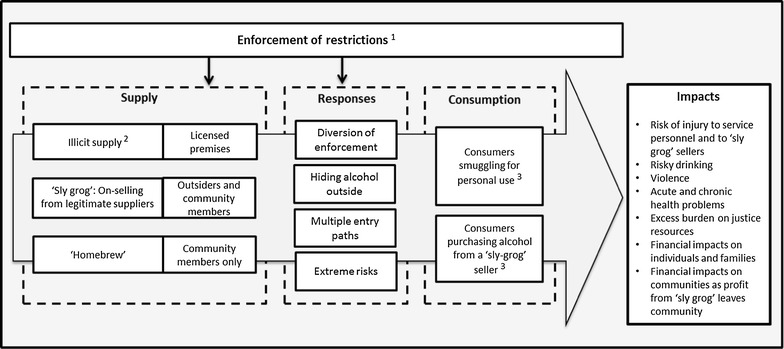
Model of illicit drinking and related impacts including relevant legislation, regulations and penalties. *1* Legislation and regulation relevant to targeting ‘sly grog’, ‘illicit alcohol’ and ‘homebrew’ In 2008, Queensland’s Parliament introduced legislative amendments proposed in the *Aboriginal and Torres Strait Islander Communities (Justice, Land and Other Matters) and Other Acts Amendment Bill (2008)* [30]. To address ‘sly grog’ specifically, this *Bill* required amendments to the *Liquor Act (1992)* adding Section *168C* [31] which made it an offence to *attempt to take liquor into* a restricted area. ‘Homebrew’ is controlled under Section *168B* of the *Liquor Act*, and *the Aboriginal and Torres Strait Islander Communities (Justice, Land and Other Matters) Act 1984* [32]. *2* Trading conditions for licensed premises in the ‘catchment’ areas By 2009, licensed premises located in the region’s mining and tourist towns, regional service centres, and urban areas became subject to ‘harm minimisation’ conditions [33]. For a total of 29 ‘catchment’ licensed premises these conditions included bans on the sale of larger containers and stronger types of liquor [25]. Licensed premises located closer to the communities were also specifically required to: keep a ‘bulk sale’ register for all takeaway sales; not sell, or have strict limitations on selling, stronger types of alcohol, and not sell alcohol to persons known to be travelling into a restricted area, i.e. a community with an AMP. *3* Penalties for breaching the AMP restrictions under *Section* *168B*, penalties vary dependent on the number of previous convictions [26]. Vehicles found carrying alcohol may be confiscated. Penalties up to AUD$22,382 currently apply to breaches of ‘homebrew restrictions. The maximum penalty for a conviction under *Section* *168C* is currently AUD$37,500

Thomas’ inductive technique was used to analyse textual information in transcribed interviews and verbatim interview notes [29]. Pertinent and impactful statements among the 542 comments about ‘illegal drinking’ were initially coded by author MF assisted by other project staff (using Nvivo 11^®^). Sub-nodes captured comments about ‘sly grog’, ‘homebrew’ ‘cost’ and ‘ease of access’ to illicit alcohol. Author AC, who was not involved in the coding, together with MF, examined the content of the project team’s detailed field notes which were completed as the data were collected, to ensure the material coded was consistent with the research team’s observations and reflections. Author MF then conducted additional coding to group the impactful statements. This permitted candidate elements for the model in Fig. 2 to be specified. Together authors MF and AC conceptualised and designed the initial model and selected a subset of the 542 comments for efficient summarization of the evidence underpinning the model.

Author JR independently examined the coded material, the detailed field notes and the limited available published and unpublished literature and media narratives about ‘sly grog’ and ‘homebrew’. Finally all authors reached consensus on the model’s proposed form (depicted in Fig. 2) and the evidence supporting it (Table 1). This iterative process helped to ensure that the model elements, and links between them, together with the underpinning evidence, were logically consistent with the project team’s collective understanding of the available information.

**Table 1:** Statements relating to the elements of the model of illicit supply and consumption of alcohol

Element	Label	Statements
Suppliers		
Legitimate sources	A	There’s no restriction on wholesaling of alcohol. So people go to [large liquor supplier] or somewhere like that in Cairns, a liquor wholesale there. They don’t even need to go, they can get [trucking company] to bring up a pallet of booze one way or the other which could be delivered to a house or a property or somewhere like where there’s no record of it what so ever. That’s then sold for astronomical rates. (Male, 50+, non-Indigenous, local community)
	B	What they [‘sly grog’ sellers] were doing was they come and stash alcohol in front garden of the house and come back at night to pick it up to take it back to [community name]. [….] They were putting bottles of rum in the car from garden. They must have went to [licensed premises] and got bottles and put down that address, and the next guy and the next guy [did the same]. Then to the [another licensed premises], [they] ended up with 23 bottles rum, 7 cartons. (Female, 25–50, Indigenous, Indigenous policy and services)
On-selling from legitimate sources ‘sly grog’	C	…..there is a bit of intel that there is sly grogging I guess you could say, someone in [regional centre] is buying a whole heap and the locals are buying off them because it’s not on registers or stuff like that which kind of makes sense, because sometimes you will have something go off here and you get the register from [regional town] and there will be like 3 or 4 cartons, and you will be like they got more than 3 or 4 cartons these guys. (Male, 25–50, non-Indigenous, Police, region 1; Male, 25–50, not-Indigenous, Government-justice)
	D	People from other communities come here purposefully to sell to [a range of communities and towns named]. Family members sell in the communities where they have families. (Female, 25–50, Indigenous, local community)
	E	It’s not only [AMP community name] people but other outside people come here (to sell grog). Not white fellas, black fellas. (Male, 50 + , Indigenous, local community)
	F	There are locals that supply it, there is white fellas that supply it to them…. (Male, 50 + , not-Indigenous, Government)
Homebrew	G	It has affected young people, with earlier age of onset of renal failure. People drink the home brew before it has fermented properly. (Focus group: Female, 25–50, Indigenous; Female, 25–50, Aboriginal; Male, 25–50, Indigenous; Government-health services)
	H	Then they get chest pains, tummy pains, diarrhoea. (Female, 25–50, Indigenous, Government-health services)
	I	When they took away the alcohol it left us with alcoholics. We (at the clinic) were worried about withdrawals. There is no rehab service (for detoxification). But no-one had withdrawals. Why? Because they made home-brew. (Female, 25–50, Indigenous, Government-health services)
Responses-challenges for Police enforcement		
Challenges for police enforcement	K	When they left, the cops were at end of street, and he said “are you sure alcohol went into the car?” I said “yes”. They rang me next day to say thanks, but it was hard to ring up, you know, ‘cos it’s my people. But when they told me 23 bottles, that’s good, they’re only paying 50 something dollars for bottle and selling for 200, 300, 400 dollars in there [AMP community]. (Female, 25–50, Indigenous, Indigenous policy and services)
	L	The police, for instance, the police switch off here at 2o’ clock in the morning, you know, you can’t get it. [….] So if they buy a couple of cartons of grog here, in trading hours, they go round to their mates place, and then through the middle of the night, after 2o’ clock, they just drive out there at will. No one’s gonna pull ‘em up. You could drive out there today and I bet you’d drive into that community without seeing a copper. They don’t have the numbers here, so you can’t blame the police ‘cos they don’t have the numbers here to do it. (Male, 50+, Indigenous, other-liquor retailer)
Diversion of enforcement	M	But they knew by 1 [o’clock] in the morning when nothing else was coming through that everyone else had been warned. By this time people had got back to [community name], they’d made the phone calls and said “Don’t come”. So they know probably about 3 of 4 o’clock that morning that another two car loads are coming in but they didn’t catch them. Because they knew, well they knew by the aftermath the next day. But those people had been warned enough to say “there’s a road block there, don’t come. We’ll let you know when the police come back into town”. (Female, 50+, Indigenous, Indigenous policy and services)
	N	There’s only one area where you can get phone reception. And they will pull up there and ring up their mates sitting out the front of the Police Station, and ask how many police cars at the station, the boys will give them the signal and if all the cars are there, they will continue. (Focus group: Female, 50+, Indigenous; Male, 25–50, not-Indigenous; Male, 25–50; Indigenous, Government-justice)
Hiding alcohol outside the restricted area	O	The Rangers find grog buried in fridges and eskies [coolers]. The grog runners bring it into the designated spots and the buyers go out and pick it up when they can. (Female, 25–50, Indigenous, local community)
	P	People are digging up holes and burying alcohol. People on mobile phones calling and telling ‘hey police coming, ditch the alcohol’. (Female, 25–50, Indigenous, local community)
Multiple entry paths	Q	….. there are so many routes into this place that they know about that we don’t… they have got a network… the police play cat and mouse, they might get one-twentieth of it I would say, if that. (Male, 50+, not-Indigenous, Government)
Extreme risks	R	And I still remember one night, there was three of us in the police vehicle. We were sitting next to the highway and we could hear a truck coming…. And so we had the lights turned off and the car turned off so we could hear. And we could hear this truck coming towards us. And all of sudden one of the constables has gone, “they’re coming with their headlights off”. So all of sudden I’ve realised they’re going to plough into us as we’re doing this road stop. So all of sudden, thanks to this constable’s quick thinking, he has turned the truck on, turned on the headlights, and thrown it into reverse,… at the same this truck was doing 120 km/h plus and is within 100 m of the front of us. So we started reversing back, this truck’s trying to do a runner. I could see it was full of people. And they’ve gone around us and they’re trying to get back to [community name]…. And they were starting to weave all over the road. Eventually, they’ve lost control and slid and thank goodness didn’t roll. And people have just piled out. I didn’t realise how many there were and the car was full of spirits and other alcohol. And aside from nearly everyone getting killed from a head on… we get into a fight with these guys, and there’s six of them and three of us….. and I’m think this is all over grog. There’s so many people who have nearly died in the last 10 min, over alcohol. (Male, 25–50, not-Indigenous, Government-justice)
Profit margin and impacts from the trade		
Profit margin	S	A five litre carton of [wine brand] is $200. Four litres is $100. (Male, <25, Indigenous, local community)
	T	…its [prohibition] just created a very lucrative black market, $350, $380 bottle of rum. (Male, 50+, not-Indigenous, Government-health)
	U	Last year or the year before there was one bottle of rum left. I think they got $850 for it. Last one during the wet. (Focus group: Female, 50+, Indigenous; Female, 50+, not-Indigenous; local community)
	V	One car seized, 25 bottles rum, 13 cartons beer, few other things. (Male, 25–50, not-Indigenous; Man 25–50, Indigenous; Government-justice)
	W	But last week alone, I think the Police got 18 bottles of rum off one flight, [unclear]… new Police Sergeant, new approach to it, did a random night time, pop out late at night they got another 27 bottles of rum. (Male, 50+, not-Indigenous, Government-health)
Impacts	X	….and noticing there is still a lot of violence happening there, even though it is meant to be a dry community, there is a lot of sly grogging happening down there, when alcohol comes to the community it gets quite volatile down there. (Male, 25–50, not-Indigenous, Other-non government)
	Y	“There is some big bucks being made [with sly grog]… they drink to get drunk because everybody else wants your grog… you got to rush the drinking so you don’t get charged. (Male, 50+, not-Indigenous, Government-health)

### Ethics

Approval was provided by the Human Research Ethics Committee (HREC) James Cook University (H4967 & H5241), the Cairns and Hinterland Health Services District (HREC/13/QCH/130–879) and Townsville and District (HREC/13/QTHS/178). Queensland Police Service Research Committee approved the research.

## Results

### Characteristics of key community members and service providers

Across the focus groups and interviews 255 community leaders, service providers and other stakeholders contributed to the information comprising the 542 comments recorded. Amongst these participants four main groups are represented.

1. *‘Local community’* 34% were community people,
2. *‘Indigenous policy and services’* 12% represented Indigenous policy or service agencies situated in the regional centers,
3. *‘Government’* 44% represented Government service agencies,
4. *‘Other’* 10% were non-Government or other interests (including liquor retailers).

Overall, participants who identified as Aboriginal and/or Torres Strait Islander (Indigenous) comprised the majority (58%) with balanced proportions of males (55%) and females (45%).

### Proposed model of illicit drinking

Figure 2 depicts the system of supply, distribution and consumption of illicit alcohol including ‘sly grog’ and ‘homebrew’ in Queensland’s communities where AMPs are in place. Figure 2 also summarises Queensland’s regulatory requirements and penalties applying to the illicit possession and consumption of alcohol in these communities.

The overall concept in Fig. 2 is that, moving left to right, illicit alcohol is supplied to community residents (consumers) from three main supply sources within a regulatory structure that demands unique policing efforts. There are specific ‘responses’ to enforcement efforts by those supplying alcohol illegally; depicted in Fig. 2. Additionally, Fig. 2 highlights the financial impacts as illicit alcohol is exchanged for cash at inflated prices, with money often leaving the local community economy. Figure 2 also lists other impacts that were frequently identified in interviews. For consumers, these include acute and chronic health issues and risky drinking behaviours which overlap with community impacts of violence and injuries and risks to local service personnel. Table 1 takes each element of the model in Fig. 2 in turn, and lists selected qualitative evidence from which the model’s components were derived. Additional interpretation and commentary is provided in the following sections with the evidence in Table 1 labelled with capital letters ‘A’, ‘B’, and so on, for easier cross-referencing. In the presentation of the results, the characteristics of participants commenting (age group, ethnicity, and the participant group represented) are listed.

### Suppliers

Three sources of supply were mentioned in interviews: (a) illicit supply of alcohol purchased from legitimate licensed sources in the towns and regional centers which is then taken into the restricted areas for consumption; (b) illicit supply by ‘sly grog’ sellers who may purchase legitimately but sell onwards to restricted area residents and (c) a small but locally significant source of ‘homebrew’ for both consumption and on-selling.

#### Alcohol purchased from legitimate licensed sources taken into restricted areas for consumption in defiance of restrictions

For some of the more remote communities, particularly those in the Queensland’s remote far north and northwest, alcohol is sourced legally from venues licensed to sell takeaway alcohol located long distances from the community. In the largest regional center (Cairns in far north Queensland where this study was headquartered), at the time of writing, there were no restrictions on consumers wishing to buy liquor in bulk. A bulk purchase of alcohol from licensed premises at long distances from AMP communities avoids the need for any record of the sale on a ‘bulk sale register’, an enforcement and monitoring tool (described in Fig. 2) required of many licensed premises located nearer to the affected communities [34]. The following quote illustrates how alcohol from such sources is known to enter the ‘sly grog’ trade:

*There’s no restriction on wholesaling of alcohol. So people go to* [large liquor supplier] *or somewhere like that in Cairns, a liquor wholesale there.* [….] *That’s then sold for astronomical rates.*

(Male, 50+, not-Indigenous, local community) The full quote is at comment ‘A’, Table 1.

 

Stockpiling of alcohol accumulated by a number of individuals who purchase small amounts from a single liquor outlet, or single purchases from several outlets, was another strategy reportedly used to avoid ‘bulk sales registers’ (see comment ‘B’, Table 1).

In some narratives, participants disclosed that community members purchase small amounts of alcohol when in regional centers for other routine purposes, e.g. shopping or visiting family. With no restrictions applying, the purchase of alcohol from legitimate suppliers at regular retail prices is not illegal, of course. However, in some situations these people can be in breach of restrictions if they return to their community with prohibited quantities and types of alcohol. Whether the alcohol is for personal consumption, or transported unknowingly, residents risk prosecution even without any subsequent on-selling or intention to do so, as Fig. 2 depicts and as regulations stipulate.

#### ‘Sly grog’: on-selling from legitimate sources

A ‘sly grog’ trade in the more remote communities, which typically have prohibition in place, is not a new phenomenon. Consistent with existing literature [35], long-term community residents reported the trade had been present since the 1980s but with demand for alcohol increasing after the 2008 tightening of restrictions. Many participants asserted that it was ‘outsiders’ who purchase, transport and supply ‘sly grog’:

*Sly*-*grog, especially mainly outsiders are selling it because they know it’s big money.*

(Female, 50+, Indigenous, local community). (Also see comments ‘C’ and ‘D’, Table 1).

However, local community residents and residents of other AMP communities are also implicated:

*For example a lot of people from* [AMP community name] *go to sell grog in* [another AMP community name] *and it’s their way of making money.*

(Focus group: two Females, 25–50, Indigenous, local community) (Also see comments ‘D’, ‘E’ and ‘F’, Table 1).

#### ‘Homebrew’

‘Homebrew’ manufacturing and consumption (Fig. 2) is not as widespread as ‘sly grog’. In a handful of communities, ‘homebrew’ is reportedly manufactured from a range of fermentable ingredients available from the local community store, e.g. yeast, fruit, fruit juice and bread:

*…. they brew it up themselves using Vegemite* [yeast extract] *and apple juice and things like that and distil it in the roofs of their houses and stuff like that and drink the stuff.*

(Male, 25–50, not-Indigenous, Government)

‘Homebrew’ is a seasonal activity in just a few communities but is of principal concern in one very isolated community. In this locality, ‘homebrew’ is produced mainly for personal consumption, often shared with family. However, bottles of ‘homebrew’ were reportedly sold for approximately $AUD20 in this community. We found no evidence that distillation was practised. Community members and service providers alike reported serious concerns about the health impacts of ‘homebrew’ mixtures, especially if isolated reports that ‘homebrew’ is occasionally fortified with other ingredients containing alcohol can be confirmed (see comments at ‘G’, ‘H’ and ‘I’, Table 1).

### Challenges for police enforcement

Police invest significant time and resources patrolling access points into restricted areas, often based on intelligence from local community members and occasionally from liquor licensees in the regional centers:

*So, for example, and the police know the windows, I know one night the* [community name] *boys got a bit of a tip off they were coming in from* [regional center]*….And the licensees will try and let people know things are happening like the* [community name] *Police. So they set up a road block, 3 cars that they got quite a stash of grog….*

(Female, 50+, not-Indigenous, Indigenous policy/services) (Also see comment ‘K’ and ‘L’, Table 1).

In making decisions about allocating their resources, Police are obliged to weigh efforts to prevent illicit alcohol reaching the community against the consequences of having it consumed in the community, consequences which can include serious domestic violence. Policing alcohol restrictions also impacts on the capacity of Police to conduct normal policing duties and other routine justice administration activities:

*They can’t deal with DV at night and be up to catch the grog runners in the day.*

(Male, 25–50, not-Indigenous, local community).

In the more remote localities, the distances involved and the limitations on staff resources magnify these challenges (see comments ‘M’ and ‘N’, Table 1).

Police also report challenges of effecting convictions of ‘sly grog’ sellers known to them. As recognised in other jurisdictions [36], Police in this study reported that in the majority of cases where individuals are apprehended with large quantities of alcohol, the necessary evidence (sworn statements, documents or other materials) implicating those individuals in any ‘sly grog’ trade often does not exist. Without such evidence, individuals charged and brought before a court can raise the defence that the alcohol was for their personal use only (i.e. a party or celebration); thus reducing the risk of a more severe penalty.

### Responses to enforcement

#### Diversion of enforcement

Organised measures, specific to each community, were reportedly used by ‘sly grog’ sellers to avoid detection. With mobile phone communications possible in all the AMP-affected communities but with service reach dependent on distance and terrain, ‘sly grog’ syndicates take advantage of the locations where reception is possible along the various access roads to communities. Informants in the community advise those transporting ‘sly grog’ of the whereabouts of Police to minimise the risk of apprehension, as the quote below illustrates:

*There’s only one area where you can get phone reception. And they will pull up there and ring up their mates sitting out the front of the Police Station*…..

 (The full quote is provided at comment ‘N’, Table 1).

Another strategy is to use decoys. For example, a vehicle containing the prohibited alcohol travels towards the restricted area behind another vehicle reducing the chances of being intercepted, e.g. at a Police road block. Placing hoax calls reporting incidents that demand a Police response is also a commonly-used strategy, e.g. reports of fighting or attempted suicides in the community and road crashes outside the community.

#### Concealing alcohol outside the restricted area

Concealing alcohol in bushland outside the restricted area affords the opportunity for customers to retrieve their own alcohol indirectly from the seller. The seller thereby avoids the risks associated with transporting the alcohol into the community for direct delivery (see comments ‘O’ and ‘P’, Table 1).

#### Multiple entry paths into remote isolated localities

Multiple transport routes and modes are possible in the region. There are numerous bush tracks to use and with communities located on the coast, some are also within a small boat’s journey of regional centers:

*If they* [Police] *are there, they will take a back track and take it around, or they will meet a boat and the boat will go up the creek and bury it in the mud, and leave a stick.*

(Focus group, Indigenous policy/services: Female, 50+, Indigenous; Male, 25–50, not-Indigenous; Male, 25–50; Indigenous). (See also comment ‘Q’, Table 1).

#### Extreme risks

In some of the more remote localities, very high-risk strategies to carry illicit alcohol into the communities were reported. For example, some bringing illicit alcohol are known to drive at night, without headlights, at speed, on the unsealed roads and bush tracks, with vehicles heavily loaded with alcohol and people. Faced with these circumstances police must avoid engagement in pursuits to reduce the prospect of vehicle roll overs, serious risk of injury or fatality, as this example suggests:

*And they were starting to weave all over the road. Eventually, they’ve lost control and slid and thank goodness didn’t roll. And people have just piled out. I didn’t realise how many there were and the car was full of spirits and other alcohol. And aside from nearly everyone getting killed from a head on … we get into a fight with these guys, and there’s six of them and three of us….. and I’m thinking this is all over grog.*

 (Male, 25–50, not-Indigenous, Government). (See comment ‘R’, Table 1 for the full description of this incident).

### Profit margin and impacts from the trade

The prices of ‘sly grog’ reported were, on average, from four to six times, and up to 11 times, its legal retail value (Table 1 comments ‘S’, ‘T’ and ‘U’). The more remote communities with total prohibition, and located furthest from regional centers, were believed by participants to have generally higher rates of profit in the ‘sly grog’ trade. The tropical ‘wet season’ limits travel and provides opportunities for ‘sly grog’ sellers to further inflate their prices as isolation is intensified. Conversely, participants noted increased ‘sly grog’ activities during the ‘dry season’ when roads are passable (information not shown).

Whisky or rum were the most frequently mentioned forms of ‘sly grog’ and the preferred types of alcohol in the trade (see comments ‘V’ and ‘W’, Table 1). These forms bring the largest profit margin for the volume involved, easy concealment for transport and ready consumer demand where rapid intoxication is desired. The quantities and value of alcohol that participants believe were typically seized (comments ‘V’ and ‘W’, Table 1) are consistent with the quantities reported seized in the media [37].

Household budgets were seen as impacted as illicit alcohol is exchanged for cash at inflated prices. As well as the impacts already described (Fig. 2), participants described surges in public and domestic violence linked with ‘sly grog’ and changed drinking behaviours towards heavier, episodic drinking:

*Yes, there’s a lot of fighting and there’s a lot of sly*-*grogging. Very often. People fight after they have alcohol in their system.*

(Two females, 25–50, Indigenous, local community) (see also comments ‘X’ and ‘Y’, Table 1).

 

## Discussion

### Summary

In communities affected by AMPs in Queensland (Australia), illicit alcohol supply and consumption is consistently reported as an issue of concern. These reports most often reference the more remote communities where total prohibition is in place. ‘Sly grog’ is the overwhelming and widespread concern while ‘homebrew’ is a persistent issue, particularly in one community and intermittently in a few others. Consistent with the limited information we have about other ‘sly grog’ markets in Indigenous Australian communities [15], illicit suppliers were driven largely by a sustained and continued demand for alcohol in combination with consumers’ willingness to pay the inflated prices. At the high profit rates reported (up to 11 times normal retail value), the potential value of the trade in these communities, where income-earning opportunities are limited, would provide a very significant motivation for its continuance.

With historical roots in experiences of the prohibition era, among First Nations populations in north America and Canada, the term ‘bootlegging’ is used to describe this same kind of activity [38, 39]. The term ‘sly grog’ has been in the Australian vernacular since early European settlement, specifically referring to the unlicensed (‘on-the-sly’) sale of diluted rum (‘grog’ in British navy slang) during the early colonial period [40]. The literature from other countries where legal controls on alcohol are used in Indigenous populations, reports significant reductions in violence and injury [4, 9, 41, 42]. However, the behaviours linked with the components of the model in Fig. 2 of ‘sly grog demand, supply and illicit consumption in response to statutory controls on alcohol appear to be unique in the literature.

### What opportunities to address ‘sly grog’ does the model of illicit alcohol suggest?

#### i. More sophisticated and comprehensive bulk sales registries that are more readily accessible to enforcement

To manage access to ‘sly grog’, more rigorous, targeted application of retailing conditions at the supply points is a strategy of central importance given the challenges of enforcing supply control at the borders of restricted areas that we have detailed. At present, a ‘bulk sales register’ [34] is the only form of takeaway sales documentation. It is paper-based and this limits its capacity to be readily accessed by enforcement. Moreover, the current regulations fail to cover licensees in a sufficiently wide catchment area since our evidence shows that ‘sly grog’ sellers are willing to travel long distances to circumvent liquor licensing conditions. A regional approach to manage takeaway sales from licensed sources would be required. Such a strategy would use the more sophisticated surveillance opportunities that electronic sales records provide compared with the limitations of paper records. Using technology, not paper, to record sales would make such information readily available to enforcement across a wide region.

In the neighbouring jurisdiction of the Northern Territory, a ‘banned drinkers register’ had the primary purpose of reducing access to alcohol among known problem drinkers [43, 44]. A similar model for ‘sly grog’ sellers in Queensland may permit more effective controls at point of sale.

#### ii. A focus on the ‘sly grog’ sellers

As indicated in Fig. 2 ‘sly grog’ sellers are charged and penalised for the same offence as a consumer transporting alcohol into a restricted area. While ‘sly grog’ sellers are liable for significant penalties upon conviction and possible imprisonment for repeat convictions [25], generally the court outcomes received have been seen as trivial and token compared to the potential profit that on-selling provides [45]. Within AMP-affected communities, these inconsistencies may have created the perception that there is limited deterrence for ‘sly grog’ sellers. Although complex, making a clearer legal distinction between ‘sly grog’ sellers and consumers could be an important opportunity to change community perceptions about these behaviours and the consequences. Threshold quantities in a person’s possession could be defined to trigger higher-level legal responses to ‘sly grog’ sellers. Consumers of ‘sly grog’ would benefit more from treatment strategies to reduce their demand for and consumption of illicit alcohol than from a severe penalty.

#### iii. Addressing demand

To be effective, alcohol supply controls must also go hand-in-hand with initiatives that address the demand for alcohol and the broad social determinants underlying alcohol misuse. Initially, in Queensland’s Indigenous communities, restrictions were designed to act as a ‘circuit breaker’ to interrupt alcohol access and to provide an environment in which to implement demand reduction strategies (rehabilitation, treatment and diversion) [23]. Demand reduction strategies are considered to be one of the most effective strategies to address alcohol misuse [46]. However, since their adoption, AMPs in Queensland have come to have a narrow focus on supply reduction. To date there are still no significant policies or programs in place to support consumers in communities affected by AMPs across Queensland. This criticism also applies elsewhere in Australia where alcohol control programs have often failed to implement designed demand reduction measures in full [16, 47].

#### iv. Community governance and mechanisms to address ‘sly grog’

While there are many non-drinkers in remote communities, there have been long-standing recommendations for effective control of harmful drinking at the local level [48]. There are several types of governance models used internationally for community-led or community-negotiated alcohol controls in Indigenous communities. Local options chosen using tribal decision making processes by First Nation and Native American groups in Canada [1, 49] and the United States [2, 4, 6, 50] are re-enforced by statute. In rural towns in Australia, regulatory authorities negotiate restrictions with Indigenous input [7, 51]. However, there is evidence that Queensland’s AMPs were imposed with little consultation [13, 27]. Community ownership and participation in decisions regarding alcohol restrictions are vital both for community authority and autonomy as well as providing a foundation for cooperative partnerships. Community-led local alcohol actions using existing coalitions supported by external government departments, rather than led by external agencies, are regarded as good practice [52]. In the Queensland setting, Government should modify its approach to legal controls on alcohol by supporting community leadership and participation in decision making regarding local restrictions [53, 54]. This could mobilise community support for enforcement efforts and reduce risky behaviours linked with ‘sly grog’.

### Limitations

The conclusions of this study should be considered preliminary, as the data it reports comprises the perceptions of a convenience sample of community leaders and service providers, and not the community populations. Moreover, the data come from a larger study which did not have illicit alcohol as its principal focus. Studies combining marketing and behavioural economics approaches are required to assess the relevance of the proposed model of illicit alcohol supply and consumption combined with epidemiological studies to test hypotheses about its impacts in the affected population (depicted in Fig. 2).

Nonetheless, it is a strength of this study that, to the authors’ knowledge, this is the first time illicit alcohol has been charted in this degree of detail from legal purchase to illegal on-selling with the impacts and challenges brought by ‘sly grog’ and ‘homebrew’ mapped in these complex circumstances across a very wide region. The qualitative approach provides a nuanced understanding of the way the demand for illicit alcohol drives the business of ‘sly grog’ and ‘homebrew’ in these localities, stimulating ideas for strategies to address the harms identified.

## Conclusions

Sly grog has serious consequences which are likely to become magnified in small remote communities. Our data indicates this is a long-standing issue that appears to be escalating. There is a strong imperative for individuals to sell illicit alcohol where there is high demand.

Although the study’s very richness also limits generalisability of specific harms and impacts to other settings [42], the structure and logic of the model we have developed may be transferable to remote Indigenous communities across Australia where similar alcohol controls have been tried. The strategies described here may assist to inform better regional management of this significant issue.

## Abbreviations

AMP: alcohol management plan
HREC: Human Research Ethics Committee
